# Evaluation of the Impact of Concentration and Extraction Methods on the Targeted Sequencing of Human Viruses from Wastewater

**DOI:** 10.1021/acs.est.4c00580

**Published:** 2024-05-01

**Authors:** Minxi Jiang, Audrey L. W. Wang, Nicholas A. Be, Nisha Mulakken, Kara L. Nelson, Rose S. Kantor

**Affiliations:** † Department of Civil and Environmental Engineering, University of California, Berkeley, California 94720, United States; ‡ Physical and Life Sciences Directorate, Lawrence Livermore National Laboratory, Livermore, California 94550, United States; § Computing and Global Security Directorates, Lawrence Livermore National Laboratory, Livermore, California 94550, United States

**Keywords:** targeted sequencing, probe-capture enrichment, human virus, wastewater-based surveillance, wastewater-based epidemiology, virus concentration, nucleic acid extraction

## Abstract

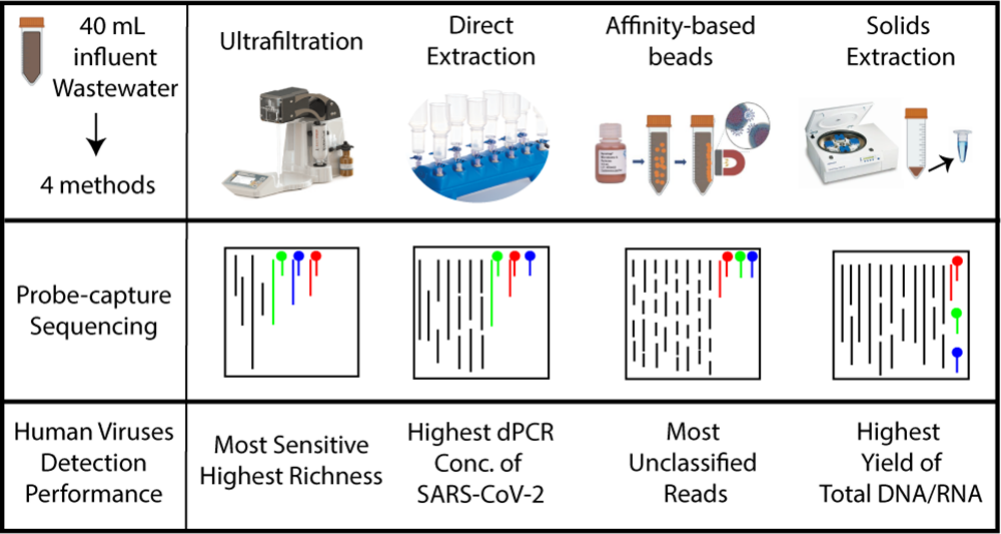

Sequencing human viruses in wastewater is challenging due to their low abundance compared to the total microbial background. This study compared the impact of four virus concentration/extraction methods (Innovaprep, Nanotrap, Promega, and Solids extraction) on probe-capture enrichment for human viruses followed by sequencing. Different concentration/extraction methods yielded distinct virus profiles. Innovaprep ultrafiltration (following solids removal) had the highest sequencing sensitivity and richness, resulting in the successful assembly of several near-complete human virus genomes. However, it was less sensitive in detecting SARS-CoV-2 by digital polymerase chain reaction (dPCR) compared to Promega and Nanotrap. Across all preparation methods, astroviruses and polyomaviruses were the most highly abundant human viruses, and SARS-CoV-2 was rare. These findings suggest that sequencing success can be increased using methods that reduce nontarget nucleic acids in the extract, though the absolute concentration of total extracted nucleic acid, as indicated by Qubit, and targeted viruses, as indicated by dPCR, may not be directly related to targeted sequencing performance. Further, using broadly targeted sequencing panels may capture viral diversity but risks losing signals for specific low-abundance viruses. Overall, this study highlights the importance of aligning wet lab and bioinformatic methods with specific goals when employing probe-capture enrichment for human virus sequencing from wastewater.

## Abstract 2

Four concentration/extraction methods combined with probe-capture sequencing of human viruses in raw wastewater were compared. Innovaprep ultrafiltration with solids removal had the best performance for human virus detection sensitivity, richness, and recovery of near-complete genomes.

## 1 Introduction

Wastewater-based epidemiology (WBE), previously employed for monitoring enteric viruses like polio,^1^ has been widely applied during the COVID-19 pandemic. In 2020, the US Centers for Disease Control and Prevention (CDC) launched the National Wastewater Surveillance System (NWSS) to build and coordinate the capacity for WBE as a component of the nationwide monitoring of SARS-CoV-2.^2^ Subsequently, groups around the world have expanded WBE to include PCR-based monitoring of known seasonal respiratory viruses including respiratory syncytial virus (RSV) and influenza A and B, and new PCR panels are expected to contribute to CDC NWSS.^3^

Unlike PCR-based virus quantification, sequencing of viruses in wastewater has the potential to monitor many human viruses at the genome level simultaneously. Reference-based amplicon sequencing using tiled panels such as ARTIC SARS-CoV-2,^4^ ARTIC HAdV-F41,^5^ Swift Normalase Amplicon Panel,^6^ or targeted amplicons like those for the VP1 or VP4 regions of enterovirus^7,8^ have enabled subtyping and tracking of circulating variants and strains, providing evidence that wastewater data align with available clinical data.^4,5^ However, amplicon-based sequencing is limited in its ability to detect novel viruses due to the challenges of degenerate primer design and multiplexing. In contrast, deep untargeted sequencing offers a comprehensive view of viral diversity in wastewater,^9−11^ but human viruses constitute a minimal fraction of the microbial nucleic acids present in wastewater, approximately 0.011% of unique reads^10^ or 0.1% of the assembled contigs.^11^ To increase sequencing coverage of human viruses and to allow the detection of divergent or novel viruses in wastewater, probe-capture enrichment panels have been adopted from clinical research.^12^ Here, probes hybridize to DNA targets in a sample, allowing downstream separation of targets from background DNA. Because probe hybridization allows more mismatches than primer binding during PCR, more divergent sequences may be enriched by probe capture, potentially including novel relatives of known viruses. Recent studies that have applied virus probe-capture panels to wastewater-derived samples reported an increase in the proportion of viral reads up to 81% compared to untargeted sequencing.^13^ Although probe-capture-based sequencing enriched human viruses, most of the recovered viral content (>80%) still consisted of bacteriophages and plant viruses.^14,15^ These findings indicate that probe-capture panels are still limited in their ability to enrich target sequences in samples with large amounts of background/nontarget sequences, suggesting that the choice of upstream sample processing method may affect the detection of human viruses.

Prior to the COVID-19 pandemic, sequencing-based wastewater virus studies relied on large-volume time-intensive concentration methods that had initially been developed to culture infectious viruses (e.g., polyethylene glycol precipitation, skim milk flocculation, ultracentrifugation, and membrane filtration). Multiple studies reported that the choice of concentration method influenced the resulting virus profiles by untargeted sequencing,^9,16^ and few studies reported any sequences from enveloped viruses. During the pandemic, the demand for rapid routine monitoring of SARS-CoV-2 led to the development and wider adoption of streamlined concentration/extraction methods with lower sampling volumes, ending with qPCR or digital PCR quantification.^17,18^ These methods included size separation (e.g., Innovaprep ultrafiltration Concentrating Pipette Select, centrifugal ultrafiltration), capture based on virus surface characteristics (e.g., Nanotrap beads, electron-negative HA membrane), and direct nucleic acid extraction (e.g., Promega Wizard Enviro large-volume extraction or extraction of wastewater solids after centrifugation). These routine monitoring methods were also used to obtain SARS-CoV-2 RNA for sequencing, with varying success^19−21^ and later extended for detection of a wider spectrum of viruses.^14,15,22−24^ To date, few studies have directly compared the effects of different methods on the success of virus probe-capture enrichment sequencing. McCall et al. compared methods with very different sampling volumes (300 μL for direct extraction and 50 mL for HA filtration) and suggested that direct extraction may yield a lower equivalent volume of viruses in the final extracted nucleic acid compared to prefiltered samples.^22^ Spurbeck et al. indirectly compared five wastewater virus concentration/extraction methods, but each was applied to wastewater samples from a different location(s). They found that Innovaprep ultrafiltration yielded the highest virus sequence recovery in untargeted RNA sequencing, although most sequences corresponded to bacteriophage.^23^ These findings highlight the potential impact of concentration/extraction methods on targeted sequencing of diverse viruses, but direct comparisons and analysis of potential biases from concentration methods on sequencing performance are needed, especially for targeted sequencing using probe-capture panels.

In this study, four wastewater virus concentration/extraction methods were selected based on their ongoing use in wastewater surveillance efforts, and the success of probe-capture enrichment sequencing was compared for each method. The wastewater input volume was held constant, and the resulting nucleic acids were enriched using the Illumina virus surveillance panel (VSP). The evaluation of methods performance included total nucleic acid (TNA) quality, unique sequence output, taxonomic composition, richness, recovered genome completeness, and sensitivity comparisons between sequencing and dPCR. Ultimately, these findings improve our understanding of wet lab approaches and their compatibility with virus probe-capture enrichment and sequencing, informing tailored responses to emerging viral threats.

## 2 Materials and Methods

### 2.1 Sample Collection

Influent wastewater was collected as 24 h composite samples on three dates: March 1st, April 19th, and April 26th, 2023, from the EBMUD wastewater treatment plant (Alameda County, CA). This facility serves approximately 700,000 people, receiving domestic and industrial wastewater. On each date, the sample was transported to the laboratory on ice, and twelve 40 mL aliquots were prepared. Bovine coronavirus (BCoV) was added to each tube as a sample processing control to assess viral RNA recovery. First, one vial of BCoV (Merck) vaccine powder was resuspended in 2 mL 0.1 mM Tris-Ethylenediaminetetraacetic acid (TE) buffer and diluted 10-fold. Each wastewater aliquot was spiked with 50 μL BCoV solution and incubated overnight at 4 °C.

### 2.2 Concentration and Extraction

In this study, four recently developed concentration and extraction methods capable of processing lower sampling volumes were employed (Figure S1): Innovaprep Concentrating Pipette Select (IP method), Nanotrap bead concentration (NT method), Promega direct extraction (PMG method), and pelleted solids direct extraction (Solids method). These methods capture viruses through diverse mechanisms and target the different portions of wastewater samples (liquid, solid, total), potentially resulting in distinct recovered virus profiles. Each method was performed on three 40 mL aliquots of wastewater per sample date, alongside a negative control consisting of 40 mL 1x phosphate-buffered saline (PBS) solution (Table S1). The Qiagen AllPrep PowerViral kit was chosen because it produces RNA and DNA needed for input to Illumina VSP and includes inhibitor removal steps critical for extraction from wastewater. Furthermore, this kit is directly compatible with liquid inputs from IP and NT (according to the respective manufacturers’ protocols) and with solids inputs. All methods resulted in 100 μL purified TNA.

#### 2.2.1 InnovaPrep Concentrating Pipette Select (IP Method)

In the IP method, 400 μL of 5% Tween 20 was added to the wastewater sample and mixed by inversion, followed by centrifugation at 7000 *g* for 10 min. The supernatant was ultrafiltered using the automatic HF Concentration Pipette (Innovaprep CP-Select) and eluted with the elution fluid (Innovaprep) to produce the viral concentrate (ranging from 160 to 882 μL, Table S1). TNA was then extracted from up to 200 μL of viral concentrate using the Allprep PowerViral DNA/RNA kit (Qiagen) and eluted in 100 μL, following the manufacturer’s liquid sample extraction protocol.

#### 2.2.2 Nanotrap Magnetic Virus Particles (NT Method)

The NT method followed the Nanotrap Microbiome A Protocol, which is optimized for virus capture and is compatible with AllPrep PowerViral DNA/RNA kit (APP-091 December 2022). Briefly, 115 μL of Nanotrap Enhancement Reagent 2 (ER2) and 600 μL of Nanotrap Microbiome A Particles (Ceres Nanosciences) were sequentially added to each sample, followed by mixing and incubation. The beads were separated from the solution on a magnetic rack and washed with 1 mL of molecular-grade water. Subsequently, the beads were again collected using the magnetic rack, supernatant was removed, and 600 μL of preheated PM1 + Beta-mercaptoethanol solution from the Allprep PowerViral kit was added. The lysis mixture was heated at 95 °C for 10 min to release nucleic acids. Beads were removed using the magnetic rack, and all supernatant was used for subsequent extraction steps using the Allprep PowerViral DNA/RNA kit liquid protocol, resulting in 100 μL of final TNA.

#### 2.2.3 Promega Wizard Environ TNA Extraction (PMG Method)

The PMG method used the commercial kit from Promega (Wizard Enviro TNA) following the manufacturer’s protocol. Briefly, 0.5 mL of protease was added to each 40 mL wastewater sample and incubated for 30 min. After centrifugation at 3000 *g* for 10 min, binding buffers and isopropanol were added to the resulting supernatant before passing it through the PureYield binding column. The bound nucleic acids were washed and then eluted in 1 mL of nuclease-free water. The eluted samples were further purified, concentrated, and eluted using the PureYield Minicolumn, resulting in a final TNA volume of 100 μL.

#### 2.2.4 Solids Centrifugation and Qiagen PowerViral AllPrep TNA Extraction (Solids Method)

In the Solids method, the 40 mL wastewater sample was centrifuged at 20,000 *g* for 10 min to pellet the solids. TNA was then extracted from 0.25 g (wet weight) of solid pellets using the Allprep PowerViral DNA/RNA extraction kit. This followed the manufacturer’s solids extraction protocol, which included a 10 min bead-beating step after the addition of PM1 and Beta-mercaptoethanol solution. The final extracted TNAs were eluted in 100 μL of nuclease-free water.

DNA and RNA concentrations were quantified using the Qubit 1X dsDNA HS Assay (Fisher Scientific) and Qubit RNA HS Assay (Fisher Scientific), respectively. Aliquots of all extracts were stored at −20 °C and quantified by dPCR within 1 week and at −80 °C for subsequent sequencing library preparation.

### 2.3 Digital PCR Quantification of SARS-CoV-2 and BCoV in the Extracted TNA

Digital PCR was performed on the QIAcuity Four Platform Digital PCR System (Qiagen). All materials and conditions are summarized in Table S2a. The reaction mixtures (Table S2b) were prepared using the QIAcuity OneStep Advanced Probe Kit (Qiagen) and loaded onto either 8.5k 24-well or 26k 24-well nanoplates (Qiagen). The positive control was linearized plasmid DNA (SARS-CoV-2) or gBlock dsDNA (BCoV) from Integrated DNA Technologies, and the negative control was nuclease-free water. See Figure S2 for examples of the partition fluorescence plots of positive and negative controls. Valid partition counts ranged from 7920 to 8269 per well for 8.5k plates and 12,548 to 25,493 per well for 26k plates. Data were analyzed using the QIAcuity Suite Software V1.1.3 (Qiagen, Germany) with automated settings for threshold and baseline, followed by manual inspection. Results were plotted using a customized Python script. dMIQE checklists^25^ are provided in Table S3. The operational limit of detection was treated as ≥3 positive partitions per well.

### 2.4 Library Preparation and Targeted Sequencing

Before library preparation, DNA and RNA qualities were measured by Fragment Analyzer with the default HS NGS Fragment 1–50 kb assay and Bioanalyzer (Agilent 2100) with the Agilent RNA 6000 Pico RNA assay, respectively. Library preparation followed the Illumina RNA Prep with Enrichment kits with modifications to total input (Illumina, San Diego, CA, USA). In brief, the mixture of purified DNA and RNA from samples collected on April 19 and April 26 was diluted with nuclease-free water such that the final concentration of RNA was ≤100 ng/μL. Dilution was not conducted for IP and NT samples due to the low RNA concentrations. The DNA and RNA from samples collected on March 1 were used directly as the input for library preparation without dilution for all concentration/extraction methods (Table S1). Next, 8.5 μL of each sample was denatured followed by first- and second-strand DNA synthesis. Tagmentation of the total double-stranded cDNA was performed using bead-linked transposons (BLT), and adapter sequences were added at the same time. The resulting fragments were purified using AMPure XP reagent and amplified to add index sequences (IDT dual indexing, P7 and P5 sequences for clustering). Libraries were quantified using the Qubit dsDNA broad-range assay kit. Enrichment was performed with the Illumina VSP Panel by pooling 200 ng of each library from three biological replicates into hybridization reactions. This step was followed by bead-based capture of hybridized probes, amplification, cleanup, and quantification of the final enriched library. After library preparation, all enriched samples were pooled in equimolar ratios (0.6 nM starting concentration) and sequenced on one lane of Illumina Novaseq 6000 SP 150PE.

### 2.5 Bioinformatics Analysis Pipeline

**Figure 1:**
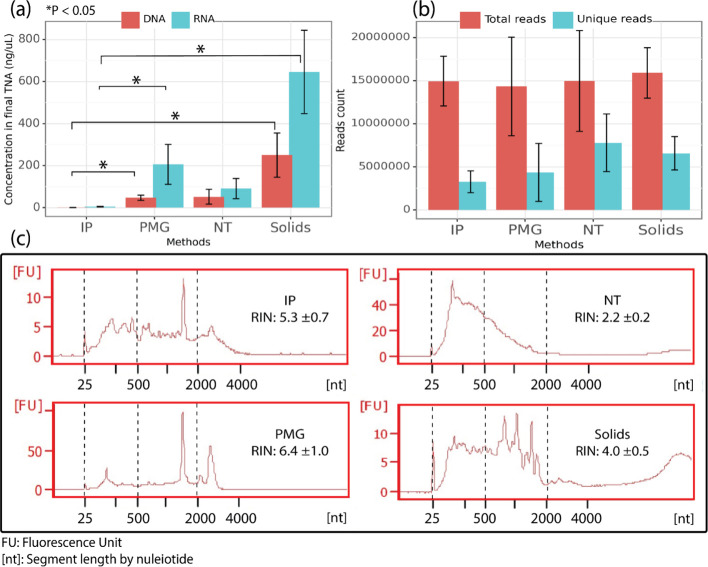
Nucleic acids and unique read counts by sample processing method. (a) Averaged concentrations of extracted DNA and RNA produced by each method (*n* = 9 samples per method); (b) averaged raw read counts and counts of unique reads after quality control, QC trimming, and deduplication in each method (*n* = 9 samples for IP, NT, and Solids, *n* = 8 for PMG); and (c) representative RNA fragment size distribution and average RNA integrity number (RIN) for each method. Note that samples were diluted before fragment analysis (IP: undiluted, NT: 25×, PMG: 25×, Solids: 200×), so *y*-axes are not comparable. Dashed lines are added to aid in visual comparison only.

**Figure 2:**
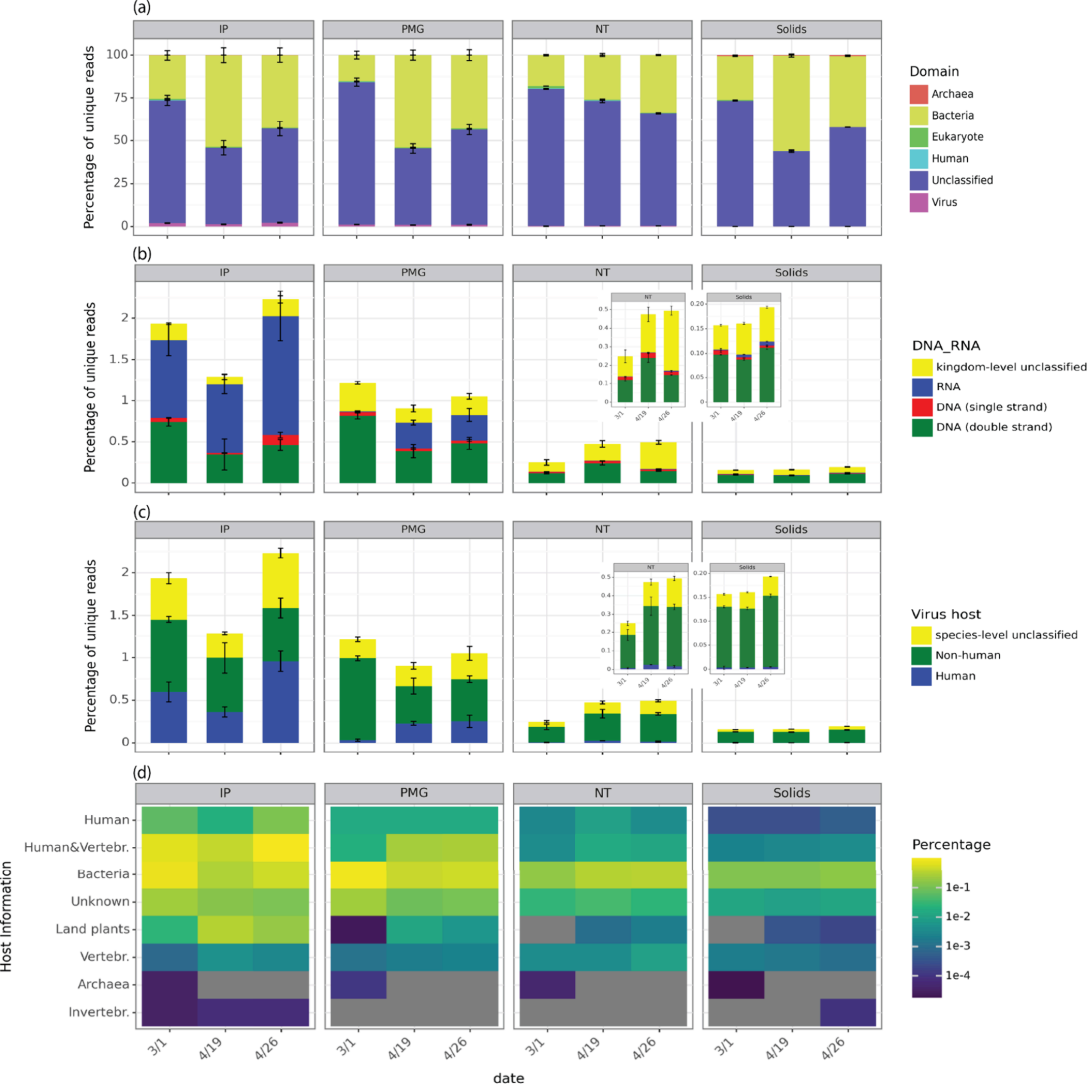
Taxonomic profiles of reads and virus hosts differed by method. (a) Domain-level classification of unique reads by Recentrifuge, with samples collected on three sampling dates and processed by four methods (*n* = 3, except Promega 4/26). “Unclassified” is the sum of reads discarded by Recentrifuge without taxonomic classification and those classified as “Root” but without a domain-level classification. “Human” represented unique reads mapped human genomes. (b) Percentages of unique reads identified as RNA, double-strand DNA, and single-strand DNA viruses based on kingdom-level virus classification. (c) Percentages of unique reads identified as virus species linked to human and nonhuman hosts in NCBI or for which species-level taxonomy was not determined. (d) Percentages of unique viral reads associated with different host categories in the NCBI Virus database. Note that “human” in (c) encompasses the categories “human and vertebrates” and “human” in (d). In (d), reads assigned to BCoV were subtracted from counts of reads assigned to “human and vertebrates” and are not displayed.

Sequence data were quality trimmed using BBduk^26^ (v 39.01) to remove adaptors and filter low-quality and short reads (with options ref = adapters ktrim = r k = 23 mink = 11 hdist = 1 qtrim = r:4:10 tpe tbo minlen = 70). Seqkit (v2.4.0) was used to deduplicate reads and summarize unique reads^27^ (Figure 1b). Human reads were filtered using bowtie2 (v2.5.1)^28^ by mapping to GRCh38.p14 (RefSeq GCF_000001405.40) and CHM13v2.0 (RefSeq GCF_009914755.1). Filtered reads were classified by Centrifuge (v1.0.4)^29^ and Recentrifuge^30^ using a decontaminated version of NCBI-nt database (NCBI release date June 5, 2023). A minimum hit length (MHL) threshold was set to 15 for Centrifuge and 40 for Recentrifuge. One sample (PMG_426_2) displayed distinct sequence properties from the other two biological replicates (Figure S3) and yielded unexpectedly low unique read counts (Table S1) likely due to unsuccessful enrichment during the library preparation. This sample was excluded from all sequencing analyses. Recentrifuge classifications at domain, kingdom, and species levels were used to compare methods (Figure 2). Determination of putative host assignments relied on the NCBI taxonomy database with manual inspection (see Supporting Information for details).^31,32^ Further species-level analyses applied a cutoff of >10 classified reads to discard low-abundance viruses.

All viral reads were extracted from each sample using rextract, and viral sequence similarities between samples were compared using MASH^33^ (v2.3). Pairwise Mash distances were calculated for the construction of the PCoA plot using the sklearn.decomposition PCA package in Python (3.10.12). A PERMANOVA test with 999 permutations was performed using the vegan package (2.6.4) in R.^34^

Reads classified by Centrifuge at the species level as severe acute respiratory syndrome-related coronavirus (taxID: 694009) were extracted and mapped to references from the GISAID database^35^ downloaded on January 2, 2024 (Table S4). The references comprised 463 complete genome sequences with high coverage and collection dates between January 1, 2023, and May 31, 2023. Mappings were filtered to <5 mismatches using reformat.sh from BBduk.^26^

Reads were assembled from each sample using SPAdes with the -meta option (v3.15.5).^36^ All virus scaffolds identified by VirSorter2 (v2.2.4)^37^ were further subjected to quality filtering, requiring a length of >1000 bp and an average coverage of >10×. These filtered assemblies were then subjected to BLASTn search against the NCBI-nt virus database, with stringent quality filters applied: > 80% identity, > 90% alignment/query length, and an e-value <1 × 10^–8^. The best hit with the highest bitscore and 100% completeness was retained for each assembled scaffold, and assembled scaffolds aligned >70% of the best hit were considered near-complete genomes (Figure S5b). The assembled near-complete genomes for JC polyomavirus were collected for phylogenetic analyses. Potential assembly errors were inspected by Integrative Genomics Viewer (IGV v 2.16.2),^38^ and genomes were recirculated by Geneious^39^ (see Supplementary Methods) before multiple sequence alignment by MUSCLE^40^ (v3.8.31) (Figure S7). The final data set included recirculated genomes, new best-hits, and the 39 JC polyomavirus reference genomes from NCBI GenBank released within two years. After identifying informative regions by GBlocks^41^ (v0.91b), IQ-Tree (v2.2.2.6)^42^ was used to select the best-fit substitution model, and the final maximum likelihood tree with branch support by 1000 ultrafast bootstrap was visualized by MEGA 11.0.^43^

### 2.6 Statistical Analysis and Data Availability

The normality of data was assessed using the Shapiro–Wilk test. Statistical differences between concentration and extraction methods were evaluated using the Kruskal–Wallis test, followed by the post hoc pairwise Dunn’s test. All statistical tests were performed using the Python package scipy.stats, and significance was determined at a 95% confidence interval (*p* < 0.05). Sequencing data for this project have been deposited in the NCBI Sequence Read Archive (SRA) under accession number: SUB13892842 and Bioproject ID: PRJNA1047067. The processed data, reproducible code, and the analysis workflow are available at https://github.com/mj2770/Wastewater-virus-surveillance.

## 3 Results and Discussion

In this study, wastewater influent was collected from a single WWTP on three dates, and viruses were concentrated and extracted by four methods: IP method (Innovaprep ultrafiltration of liquid portion paired with a small-volume extraction kit), NT method (Nanotrap beads-based affinity capture performed on total influent paired with small-volume extraction kit), PMG method (Promega large-volume direct extraction), and Solids method (centrifugation paired with small-volume extraction kit). The resulting 36 samples (12 samples in biological triplicate) were processed using the Illumina VSP panel employing probe-capture enrichment. Following the initial analysis, an outlier sample was identified, indicating unsuccessful library preparation (see Materials and Methods), and this sample was excluded from all analyses.

### 3.1 Sample Quality and Sequence Data

The DNA and RNA generated using the four methods differed in concentration (Kruskal–Wallis test *p* = 2 × 10^–6^ and 7 × 10^–7^, respectively), fragment size distribution, and RNA integrity (ANOVA test *p* = 1 × 10^–13^). The Solids method consistently resulted in yields that were higher than other methods for both DNA and RNA (Figure 1a), while the IP method, which includes a solids removal step, resulted in significantly lower total DNA and RNA yield compared to Solids and PMG (Figure 1a, IP vs Solids DNA *p* = 3 × 10^–7^, IP vs PMG DNA *p* = 0.02, IP vs Solids RNA *p* = 3 × 10^–7^, IP vs PMG RNA *p* = 0.004). This lower yield could also be due to limited sample inputs. Specifically, we input 40 mL wastewater into all methods for consistency in method comparison; however, the IP ultrafilter concentrate volume and solids pellet mass exceeded the input allowed by the extraction kit. Consequently, the effective volumes processed were less than 40 mL for IP (16.3 ± 13 mL) and Solids samples (18.93 ± 5.04 mL) (Table S1). Future users could optimize each method to maximize virus recovery (e.g., increase the effective volume of wastewater processed or decrease IP elution volumes) and compare different small-volume extraction kits to AllPrep PowerViral.

All methods yielded a higher concentration of RNA than DNA, but the resulting ratios of RNA:DNA varied significantly (Kruskal–Wallis test *p* = 0.002) across methods from 2.0 ± 0.7 (for NT) to 4.3 ± 1.6 (for PMG). Unlike the other methods, shorter RNA fragments were observed with the NT method and 16S rRNA and 23S rRNA were absent, perhaps accounting for the low RNA:DNA ratio. The lack of rRNA may be due to the exclusion of bacteria by the nanotrap hydrogel particle shells, which have specific pore sizes and are chemically modified to prevent the entry and capture of large or nontargeted particles.^44^ Although viral RNA integrity is not discernible from the RNA Integrity Number (RIN) alone, the highest RIN was observed with the PMG method (6.4 ± 1.0, Figure 1c), which suggested that more intact prokaryotic RNA was preserved with the PMG method.

After sequencing 36 samples and removal of one sample due to unsuccessful enrichment (PMG_426_2), a total of 527 million reads were generated, averaging 15.05 ± 4.37 million reads per sample (Figure 1b). The removal of PCR duplicates reduced read counts by over 50% for all samples. As the IP method produced the lowest RNA and DNA input concentrations, it was not surprising that after quality trimming and deduplication, these samples also retained significantly fewer unique reads (3.3 ± 1.3 million, Figure 1b) compared to samples from the Solids and NT methods (IP vs NT *p* = 0.005, IP vs Solids *p* = 0.04). Nonetheless, the count of unique reads was not clearly related to the DNA and RNA concentrations, perhaps due to the dilution of nucleic acids (Table S1) before library preparation, and the multiple amplification and equimolar pooling steps during library preparation.

### 3.2 Taxonomic Classification and Virus Composition Similarity

Over 40% of unique reads were not taxonomically classified by Recentrifuge at the domain level with the selected MHL across all methods, and most classified reads were assigned to the domain bacteria (ranging from 25.84 ± 6.81 to 40.88 ± 13.13%, Figure 2a). It is likely that a larger proportion of unique reads would have received an assigned taxonomy at a lower classification stringency; however, such low-confidence assignments have the potential to introduce substantial noise to downstream assessments. Future functionalization of these platforms will require tuning of these stringency thresholds for the desired application,^45^ balancing classification sensitivity with assignment confidence. These findings could also reflect the current limitations of reference-based classifiers and limited enrichment of targets using probe-capture, irrespective of the concentration and extraction methods employed.

The percentage of reads classified as viral ranged from 0.17 ± 0.02% (Solids) to 1.82 ± 0.46% (IP) of unique reads across different methods (Figure 2b), surpassing the reported <0.011% in untargeted sequencing.^9^ The IP samples yielded significantly higher percentages of viral reads than Solids and NT (1.82 ± 0.46%, Figure 2b, IP vs Solids *p* = 8 × 10^–7^, and IP vs NT *p* = 0.004), followed by the PMG samples (1.06 ± 0.18%, Figure 2b, PMG vs Solids *p* = 0.002). Additionally, the IP method concentrated significantly more RNA viruses (Figure 2b) and viruses associated with human and/or vertebrate hosts than NT and Solids methods (0.64 ± 0.27% human viruses in total unique reads from IP, Figure 2c,d, IP vs NT *p* = 0.002, IP vs Solids *p* = 1 × 10^–6^). The IP and PMG methods incorporated a solids removal step after attempting to release solid-associated viruses by adding 5% Tween 20^46^ or protease, respectively.^47^ These steps not only prevent clogging during sample processing but also strike a balance between eliminating solid-associated nonviral microorganisms like bacteria and attempting to retain viruses. As a result, a notably lower ratio of classified bacterial reads to classified viral reads was observed in IP and PMG samples (25 ± 14:1 and 38 ± 24:1, respectively) in comparison to Solids and NT samples (241 ± 83 and 66 ± 12, respectively) (IP vs NT *p* = 0.04; IP vs Solids *p* = 1 × 10^–5^; PMG vs Solids *p* = 0.0006). In NT and Solids samples, most viral reads were associated with bacterial hosts, based on the NCBI taxonomy database (Figure 2d). This finding is consistent with the high fraction of DNA viruses in those samples (Figure 2b), as most bacteriophages are DNA viruses.^48^

**Figure 3:**
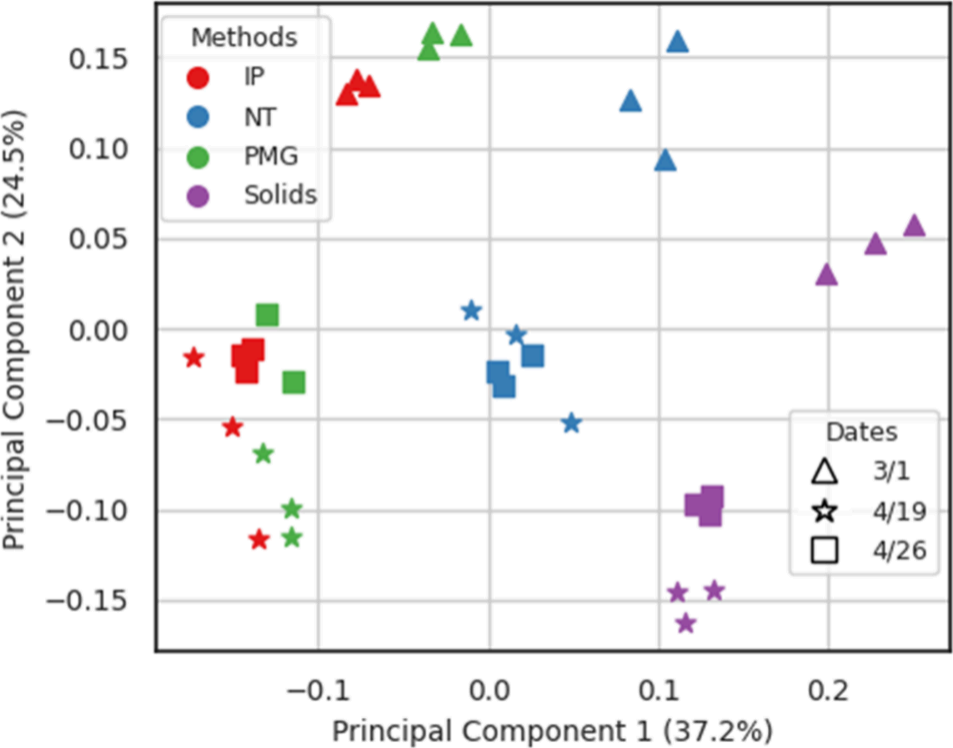
Viral sequence composition was influenced by wastewater virus concentration/extraction methods and sample date. Principal component analysis (PCoA) plot was generated using the MASH distance, which was calculated based on sequence similarity among all reads classified as viral by Centrifuge. Different methods are represented by colors, and different sampling dates are represented by shapes.

To compare virus composition across the four sample preparation methods, reads classified as viral by Recentrifuge were extracted from each sample, and MASH was used to assess pairwise sequence similarity. In a principal component analysis (PCoA) using MASH distances, triplicate samples clustered together (PERMANOVA test *p* = 0.985), while all samples were separated by concentration/extraction methods along the first principle component (PC1) (37.2% of the variation, Figure 3, PERMANOVA test *p* = 0.001). Specifically, IP and PMG samples clustered together, while NT and Solids samples were distinct (Figure 3). The predominance of bacteriophage in both NT and Solids samples likely contributed to their differentiation from the other two methods. Samples were separated by sampling dates along the second principle component (PC2) (24.5% of the variation, Figure 3, PERMANOVA test *p* = 0.001), with samples from March 1, 2023, differing from those collected on April 19 and April 26. This differentiation was observed consistently across all four methods. These temporal shifts in virus composition may suggest a temporally variable metavirome composition in wastewater, potentially influenced by changes in circulating viruses^8,49,50^ and changing wastewater conditions, such as flow rate, total suspended solids (TSS), total organic compounds (TOC), and the abundance of antagonistic microorganisms.^51,52^ Similarly, virus sequence diversity may be impacted by differences in wastewater sources, although a single wastewater source was tested in this study.

### 3.3 Human Virus Species Richness and Composition

PMG and IP methods yielded higher species-level richness of total viruses detected with >10 reads (241 and 176 viruses, respectively) and human viruses (20 and 26, respectively) compared to NT and Solids (Figure S4a), although total read depth was similar for all samples (Figure 1b, *p* = 0.44). Thus, removing solids after releasing solid-associated viruses did not compromise the richness of detected human viruses. Conversely, including solids produced lower species-level diversity. Of the 66 virus “groups” of high public health significance listed as targets in the Illumina VSP panel (Table S5), IP samples detected members of 11 (Figure S4a). These included human coronavirus-OC43 (hCoV-OC43), adenovirus, astrovirus, aichivirus, enterovirus, norovirus, coxsackievirus, rotavirus, salivirus, and sapovirus, as well as mpox (Figure S4b), though the exact list of species and strains used by Illumina for probe design is proprietary; we note that enteroviruses are a diverse group which contains coxsackieviruses, while hCoV-OC43 is a subspecies level category.

**Figure 4:**
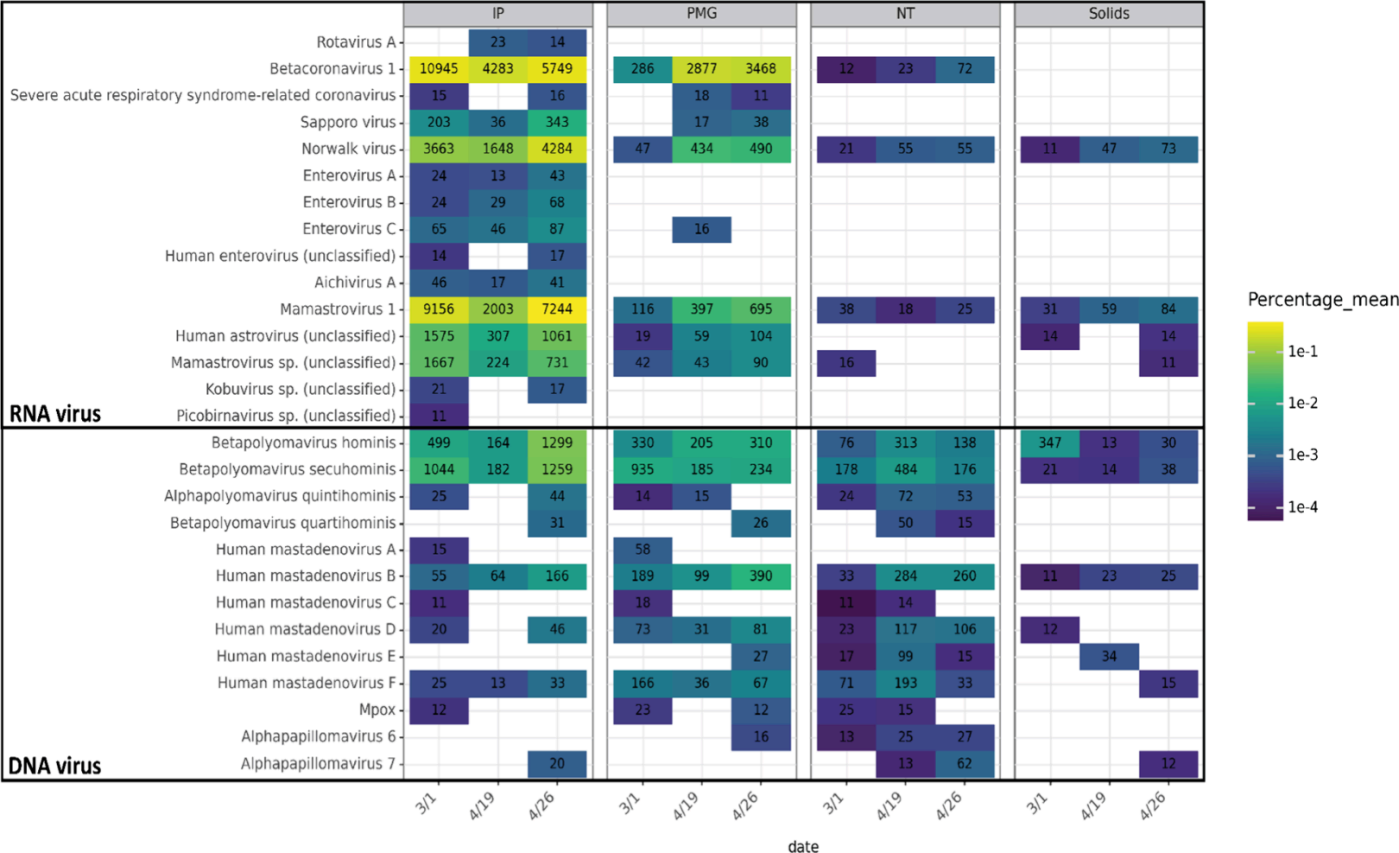
Relative abundance of human virus species in each sample. Cell color indicates the average percent relative abundance of each virus species in total unique reads across triplicate samples, based on Recentrifuge read classification. Species with fewer than an average of 10 reads per sample are not shown. Text in each cell indicates the average read counts assigned to the species for each sample. Viruses are grouped by genome type. NCBI taxIDs corresponding to species without names (e.g., “sp.”) are appended with “(unclassified)” (see Supplementary Methods). Note that Betacoronavirus 1 includes the spike-in bovine coronavirus (BCoV).

All human virus species detected (>10 reads per species) in at least one sample were compared across the four methods (Figure 4). Some viruses were consistently detected by all methods, including human polyomavirus, mastadenovirus, mamastrovirus 1, and norwalk virus, which were included in the VSP probe set and are known to be shed at high concentrations in human waste.^5,9,10,13,22,49,53−56^ RNA virus species, including severe acute respiratory syndrome-related coronavirus, sapporo virus, and enteroviruses, were not detected in NT and Solids samples. Different trends were also observed among virus species within the same genus. For instance, human mastadenovirus B, D, and F were detected in all samples, while human mastadenovirus A, C, and E were not detected in certain samples (Figure 4). This variability suggested that the detection of low-abundance species might be stochastic. No arthropod-transmitted viruses (e.g., dengue, chikungunya), bloodborne viruses (e.g., hepatitis virus and human immunodeficiency virus), or hemorrhagic fever-related viruses (e.g., lassa mamarenavirus, junin virus, etc.) were detected, despite their inclusion in the probe panel. Mpox, detected intermittently in wastewater since the outbreak in 2022,^57,58^ was detected at low levels in IP, PMG, and NT samples.

### 3.4 Potential of Recovering Near-Complete Human Virus Genomes

Seven near-complete human virus genomes were assembled from IP samples, the most from any concentration/extraction method (Figure S5b). This aligned with the high numbers of total virus and human virus reads in these samples (59,965 ± 28,180 and 20,242 ± 9294, respectively, Table S1). No near-complete human virus genomes were obtained from Solids-extracted samples (Figure S5b) likely due to insufficient reads for total viruses and human viruses (11,043 ± 2720 and 213 ± 99, respectively, Table S1). These results highlight the need to better characterize the minimum sequencing depth in relation to the proportion of viral reads required for the assembly of high-quality virus genomes.

JC polyomavirus composite genomes were assembled in samples from three concentration/extraction methods (IP, PMG, and NT) and multiple replicates (Figure S5b). The recovery of JC polyomavirus genomes is perhaps unsurprising given that approximately 40% of the population sheds the virus through urine.^53^ Also, as a nonenveloped DNA virus with a circular genome, JC polyomavirus is highly resistant to environmental stress and exonuclease activity.^9^ Ten scaffolds classified as near-complete JC polyomavirus genomes were used for phylogenetic analysis. At least one subtype of JC polyomavirus 3 was present (Node 1353 NT_301_1), affiliated with clades from South Africa (Figure S8). Although other scaffolds were clustered together, they exhibited relatively low node support values (<50); likely several of these scaffolds represent the same JC polyomavirus population in replicate wastewater samples, with variations in the composite assembly. These results, and those from other recent studies,^24^ demonstrated that probe-capture enrichment can yield whole genomes of high-abundance viruses for phylogenetic analysis, which may be useful for identifying novel virus strains in the future.

### 3.5 Comparison of SARS-CoV-2 and BCoV Detection between dPCR and Targeted Sequencing Using the VSP Panel

**Figure 5:**
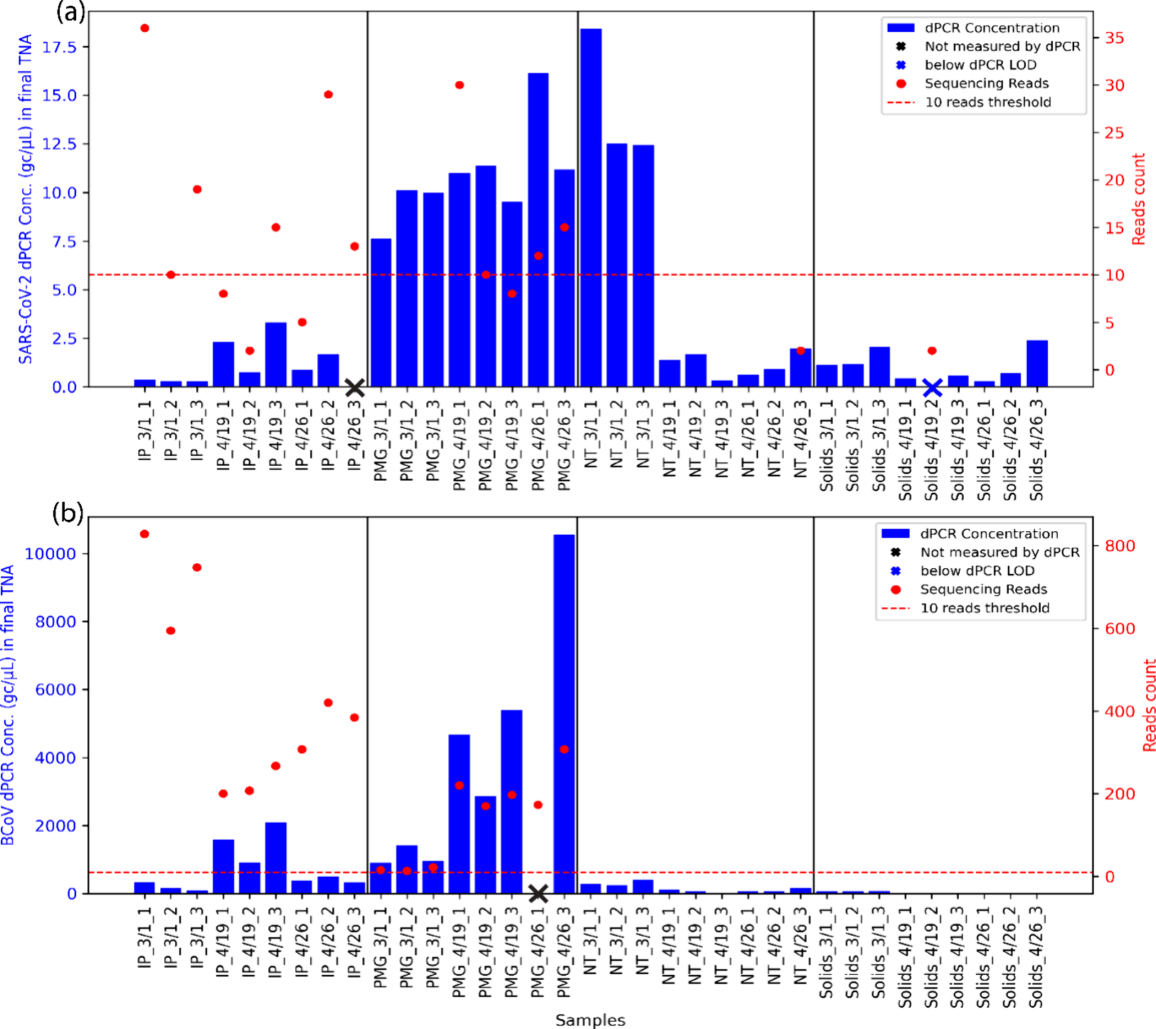
Detection sensitivity comparison between dPCR and reads-based classification of sequencing results. (a) SARS-CoV-2 detection comparison; (b) BCoV detection comparison. Blue bars on the left *y*-axis represent the virus concentration measured by dPCR in the final TNAs eluted in 100 μL after each extraction. Samples with dPCR concentration below the operational limit of detection are shown with a blue “×”, and samples without measurement are labeled with a black “×”. Red points on the right *y*-axis represent the count of unique reads mapped to SARS-CoV-2 or classified by Recentrifuge as BCoV. Note that, where measured, BCoV was detected in all samples (≥3 positive partitions), although not always visible in the plot. The dashed red line at 10 reads indicates the operational limit of detection of sequencing, as used elsewhere in the analysis.

Based on the quantification of endogenous SARS-CoV-2 and the spike-in BCoV in the final extracted nucleic acids, dPCR demonstrated higher detection sensitivity compared to sequencing across all methods (dPCR: SARS-CoV-2 33/34 detected, BCoV 34/34 detected; sequencing: SARS-CoV-2 10/35 detected, BCoV: 17/35 detected). The detection threshold for dPCR was set at ≥3 positive partitions, and the sequencing detection threshold was >10 reads. Notably, for SARS-CoV-2, many of our observations were near these thresholds (Table S6). Additionally, while BCoV is not included in the VSP probe set, the closely related Betacoronavirus 1 strain hCoV-OC43 is in VSP, potentially allowing enrichment of BCoV during probe capture. Despite differences in the effective volumes processed (see Section 3.1 and Table S1), generally, IP and PMG methods led to higher SARS-CoV-2 and BCoV concentrations in the purified TNA and in unique read counts. Meanwhile, NT and Solids methods yielded low concentrations by dPCR and no detection by sequencing (Figure 5). However, within the IP and PMG samples, the concentrations measured by dPCR did not directly correspond to SARS-CoV-2 and BCoV virus read counts (Figure 5). This could be due to the fact that probe-capture sequencing results are impacted by the ratio of targeted viruses to nontargeted background,^59^ which varied across different wastewater concentration methods and sampling dates (Figure 2). Meanwhile, dPCR measurements are unlikely to be impacted by nontarget background sequences and hence provide more accurate concentrations of viruses in wastewater. The performance differences between concentration methods in dPCR and sequencing suggest that separate methods may be best for dPCR (e.g., PMG) and sequencing (e.g., IP).

### 3.6 Implications and Limitations

Removing wastewater solids prior to extraction, but after treating the sample with either Tween 20 (IP method) or protease (PMG method), resulted in higher overall detection of human viruses via probe-capture sequencing and a higher ratio of virus-to-bacterial sequences (Figures 2, 4, and 5 and Section 3.2). In support of this observation, the ratio of target-to-nontarget in the input nucleic acids has previously been shown to affect the success of probe-capture sequencing.^59^ Accordingly, measurement of this ratio (via dPCR and/or Qubit) before library preparation and enrichment might be a useful predictor for the success of sequencing.^61^ Additional parameters of interest include target virus concentration, nucleic acid integrity, fragment size distribution, and nontarget sequence composition. Future studies should statistically compare these evaluation metrics with the final sequencing performance on a larger number of samples.

Beyond the concentration and extraction method, the sensitivity of probe-capture sequencing will likely vary with the probe panel selected and the seasonal fluctuations in wastewater microbial composition. In the present study and other studies using broad virus capture panels,^22,49^ human virus sequences were predominantly enteric virus targets present in the panel, such as mamastrovirus, and the detection of SARS-CoV-2 was limited. However, several studies using the narrower RVOP panel found remarkably high coverages of SARS-CoV-2, surpassing other respiratory human viruses included in the panel.^14,15,50^ Another study also found that the share of sequences derived from SARS-CoV-2 was higher when using the Respiratory Virus Oligo Panel (RVOP) than the broader Respiratory Pathogen ID/AMR Panel (RPIP), which included other high-abundance targets.^60^ Although this high sequencing recovery of SARS-CoV-2 may have been partially due to the higher prevalence at the sampling time, future work is needed to design and benchmark custom probe panels that balance target diversity and sequencing sensitivity for early detection of emerging virus strains.^61^

The results presented here were limited to samples from a single wastewater treatment plant over two months, two nucleic acid extraction methods, and one probe panel (as discussed above). Future work should address whether spatial and seasonal variations in wastewater physicochemical characteristics and wastewater virus concentrations differentially affect each concentration/extraction method. Comparisons of different extraction methods may also be valuable, as extraction affects the overall sensitivity of sequencing by influencing the degree of viral lysis and integrity of the resulting nucleic acids.^16^ Finally, downstream processing steps such as the use of DNase treatment during RNA extraction or rRNA depletion might be expected to improve the recovery of human RNA viruses by reducing nontarget nucleic acids.

## Supporting Information Available

All 36 samples’ processing includes concentration/extraction, quality control, and raw sequencing data QC trimming/deduplication statistics; GISAID SARS-CoV-2 reference genome accession numbers; and comparison of dPCR and sequencing reads-based classification of SARS-CoV-2 and BCoV (XLSX)
Primers, probes, and cycling parameters for RT-dPCR quantification and reaction mixtures for 8.5k and 26k 24 wells nanoplates; dMIQE checklist for RT-dPCR experiments; 66 virus targets of high public health significance in the Illumina VSP panel; schematic description of key steps in each concentration and extraction method; partition fluorescence plots of positive and negative control; clustering of all samples by PCoA plot based on the calculated MASH distance of virus sequences classified by centrifuge; the richness of detected virus species at the species level; the relative abundance of the detected viruses included in the VSP panel; assessment of assembly quality based on N50 and total assembly length; count of near-complete virus genomes assembled; representative assembly visual inspection by Integrative Genomics Viewer; dotplots of assembled putative JC polyomavirus scaffolds with repeated regions at the beginning and the end of the sequence; maximum likelihood phylogenetic tree of assembled JC polyomavirus scaffolds; virus-host classification and validation; DNA and RNA virus classification; human virus species classification; and phylogenetic analysis of JC polyomavirus (PDF)
