# Supplementary material for: Evaluation of the Impact of Concentration and Extraction Methods on the Targeted Sequencing of Human Viruses from Wastewater
Source: Environ Sci Technol. 2024 May 1;58(19):8239–50. doi: 10.1021/acs.est.4c00580 (PMC11097627; doi:10.1021/acs.est.4c00580)
Supplement: Supplementary file 2 — es4c00580_si_002.pdf [file es4c00580_si_002.pdf]

Supporting Information

Evaluation of the impact of concentration and extraction methods on the targeted sequencing of human viruses from wastewater

Minxi Jiang[a], Audrey L.W. Wang[a], Nicholas A. Be[b], Nisha Mulakken[c], Kara L. Nelson[a], Rose S. Kantor[a]*

a. Department of Civil and Environmental Engineering, University of California, Berkeley, CA, 94720, USA
b. Physical and Life Sciences Directorate, Lawrence Livermore National Laboratory, Livermore, CA, 94550, USA.
c. Computing and Global Security Directorates, Lawrence Livermore National Laboratory, Livermore, CA, 94550, USA

*Corresponding author: Rose S. Kantor rkantor@berkeley.edu

Summary:

Total pages: 21

Total supplementary tables: 6

Total supplementary figures: 8

Total supplementary methods: 4

Supplementary Tables:

Table S1. All 36 samples' processing includes concentration/extraction, quality control, and raw sequencing data QC trimming/deduplication statistics

Table S2. (a) Primers, probes, and cycling parameters for RT-dPCR quantification; (b) Reaction mixtures for 8.5k and 26k 24 wells nanoplates

Table S3. dMIQE checklist for RT-dPCR experiments

Table S4. GISAID SARS-CoV-2 reference genome accession numbers

Table S5. 66 virus targets of high public health significance in the Illumina VSP panel

Table S6. Comparison of dPCR and sequencing reads-based classification of SARS-CoV-2 and BCoV

Supplementary Figures:

Figure S1. Schematic description of key steps in each concentration and extraction method

Figure S2. Partition fluorescence plots of positive and negative control

Figure S3. Clustering of all samples by PCoA plot based on the calculated MASH distance of virus sequences classified by Centrifuge

Figure S4. (a) The richness of detected virus species at the species level; (b) The relative abundance of the detected viruses included in the VSP panel

Figure S5. (a) Assessment of assembly quality based on N50 and total assembly length; (b) Count of near-complete virus genomes assembled

Figure S6. Representative assembly visual inspection by Integrative Genomics Viewer (IGV)

Figure S7. Dotplots of assembled putative JC polyomavirus scaffolds with repeated regions at the beginning and the end of the sequence

Figure S8. Maximum likelihood phylogenetic tree of assembled JC polyomavirus scaffolds

Supplementary Methods

- Virus-host classification and validation
- DNA and RNA virus classification
- Human virus species classification
- Phylogenetic analysis of JC polyomavirus

Table S1: See excel file

Table S2:

(a) Primers, probes, and cycling parameters for SARS-CoV-2 dPCR quantification

Targeted Virus	Assays	Oligonucleotides sequences (5' - 3')<br><br>(purchased from IDT)	Cycling parameters	References
SARS-CoV-2	N1	F: GACCCCAAAATCAGCGAAAT<br><br>R: TCTGGTTACTGCCAGTTGAATCTG<br><br>P: FAM-ACCCCGCATTACGTTTGGTGGACC	50 °C for 40 min for RT;<br>95 °C for 2 min;<br>45 cycles of 95 °C for 5 s;<br>60 °C for 30 s	US CDC, 2020
Bovine coronavirus	BCoV	F: CTGGAAGTTGGTGGAGTT<br><br>R: ATTATCGGCCTAACATACATC<br><br>P: HEX-CCTTCATATCTATACACATCAAGTTGTT		Decaro, 2008*

* Decaro, Nicola et al. “Detection of bovine coronavirus using a TaqMan-based real-time RT-PCR assay.” Journal of virological methods vol. 151,2 (2008): 167-171. doi:10.1016/j.jviromet.2008.05.016

(b) Reaction mixtures for 8.5k and 26k 24 wells nanoplates

Plate types	Total reaction volumes (μL)	Mastermix (μL)	Primer and probe mix (μL)<br><br>400 nM of forward primer, 400 nM of reverse primer, 200 nM of probe	Nuclease free water (μL)	Samples/negative controls/standards (μL)	Reverse transcriptase (μL)
8.5k 24 wells	12	3	0.6	5.28	3	0.12
26k 24 wells	40	10	2	17.6	10	0.4

Table S3: dMIQE checklist for RT-dPCR experiments

ITEM TO CHECK	PROVIDED	COMMENT
**1. SPECIMEN**		
Detailed description of specimen type and numbers	**Y**	See Methods
Sampling procedure (including time to storage)	**Y**	See Methods
Sample aliquotation, storage conditions and duration	**Y**	See Methods
**2. NUCLEIC ACID EXTRACTION**		
Description of extraction method including amount of sample processed	**Y**	See Methods
Volume of solvent used to elute/resuspend extract	**Y**	See Methods
Number of extraction replicates	**Y**	See Methods
Extraction blanks included?	**Y**	See Methods
**3. NUCLEIC ACID ASSESSMENT AND STORAGE**		
Method to evaluate quality of nucleic acids	**Y**	See Methods
Method to evaluate quantity of nucleic acids (including molecular weight and calculations when using mass)	**Y**	See Methods
Storage conditions: temperature, concentration, duration, buffer, aliquots	**Y**	See Methods

Clear description of dilution steps used to prepare working DNA solution	**Y**	See Methods
**4. NUCLEIC ACID MODIFICATION**		
Template modification (digestion, sonication, pre-amplification, bisulphite etc.)	**N**	N/A
Details of repurification following modification if performed	**N**	N/A
**5. REVERSE TRANSCRIPTION**		
cDNA priming method and concentration	**Y**	See Methods
One or two step protocol (include reaction details for two step)	**Y**	See Methods
Amount of RNA added per reaction	**Y**	See Methods
Detailed reaction components and conditions	**Y**	See Methods
Estimated copies measured with and without addition of RT*	**N**	No need
Manufacturer of reagents used with catalogue and lot numbers	**Y**	See Methods
Storage of cDNA: temperature, concentration, duration, buffer and aliquots	**N**	N/A
**6. dPCR OLIGONUCLEOTIDES DESIGN AND TARGET INFORMATION**		

Sequence accession number or official gene symbol	**Y**	Table S2
Method (software) used for design and in silico verification	**N**	N/A
Location of amplicon	**N**	N/A
Amplicon length	**Y**	Table S2
Primer and probe sequences (or amplicon context sequence)**	**Y**	Table S2
Location and identity of any modifications	**N**	N/A
Manufacturer of oligonucleotides	**Y**	Table S2
**7. dPCR PROTOCOL**		
Manufacturer of dPCR instrument and instrument model	**Y**	See Methods
Buffer/kit manufacturer with catalogue and lot number	**Y**	See Methods
Primer and probe concentration	**Y**	See Methods
Pre-reaction volume and composition (incl. amount of template and if restriction enzyme added)	**Y**	See Methods
Template treatment (initial heating or chemical denaturation)	**N**	N/A
Polymerase identity and concentration, Mg++ and dNTP concentrations***	**N**	N/A

Complete thermocycling parameters	**Y**	Table S2
**8. ASSAY VALIDATION**		
Details of optimisation performed	**N**	N/A
Analytical specificity (vs. related sequences) and limit of blank (LOB)	**N**	N/A
Analytical sensitivity/LoD and how this was evaluated	**Y**	See Methods
Testing for inhibitors (from biological matrix/extraction)	**N**	N/A
**9. DATA ANALYSIS**		
Description of dPCR experimental design	**Y**	See Methods
Comprehensive details negative and positive of controls (whether applied for QC or for estimation of error)	**Y**	See Methods
Partition classification method (thresholding)	**Y**	See Methods
Examples of positive and negative experimental results (including fluorescence plots in supplemental material)	**Y**	Figure S1
Description of technical replication	**N**	No technical replicates
Repeatability (intra-experiment variation)	**N**	N/A
Reproducibility (inter-experiment/user/lab etc. variation)	**N**	N/A

Number of partitions measured (average and standard deviation)	**Y**	See Methods
Partition volume	**N**	N/A
Copies per partition (λ or equivalent) (average and standard deviation)	**N**	N/A
dPCR analysis program (source, version)	**Y**	See Methods
Description of normalisation method	**N**	N/A
Statistical methods used for analysis	**Y**	See Methods
Data transparency	raw data available on request:	

Table S4: see excel file

Table S5: 66 targeted viruses included in the Illumina VSP panel. The Illumina website does not provide a complete list of 203 strains under these 66 viruses, and the naming convention is a mix of genus, species, and sub-species levels. To track the detection of targeted viruses by each concentration/extraction method, the 66 virus names were manually checked against the NCBI taxonomy. Exact matches were recorded with virus name and taxID. For names not exactly matching NCBI taxonomy, potentially included viruses were recorded with NCBI names, taxID, and ranks under the Illumina virus names.

Illumina Name	NCBI Name	taxID	Rank	Symptom type
Adenovirus	Mastadenovirus	10509	genus	Respiratory/Cardiopulmonary
Aichivirus	Aichivirus A	72149	species	Enteric
	Aichivirus B	194965	species	Enteric
	Aichivirus C	1298633	species	Enteric
	Aichivirus D	1897731	species	Enteric
	Aichivirus E	1986958	species	Enteric
	Aichivirus F	1986959	species	Enteric
Astrovirus	Astroviridae	39733	family	Enteric
Chapare virus	Chapare virus	3052302	species	Hemorrhagic fever
Chikungunya virus	Chikungunya virus	37124	species	Anthropod tranmitted/febrile tropical
Coronavirus-229E	Coronavirus 229E	11137	species	Respiratory/Cardiopulmonary
Coronavirus-HKU1	Human coronavirus HKU1	290028	species	Respiratory/Cardiopulmonary
Coronavirus-OC43	Human coronavirus OC43	31631	no rank	Respiratory/Cardiopulmonary
Coronavirus-NL63	Coronavirus NL63	277944	species	Respiratory/Cardiopulmonary
Coxsackievirus	Coxsackievirus	12066	species	Enteric
	Coxsackievirus A9	12067	no rank	Enteric
	Coxsackievirus A22	42783	no rank	Enteric
	Coxsackievirus A19	42778	no rank	Enteric
	Coxsackievirus A1	42779	no rank	Enteric
	Coxsackievirus A4	42785	no rank	Enteric
	Coxsackievirus B4	12073	no rank	Enteric

	Coxsackievirus B5	12074	no rank	Enteric
	Coxsackievirus A6	86107	no rank	Enteric
Crimean-congo haemorrhagic fever virus	Crimean-congo haemorrhagic fever virus	3052518	species	Hemorrhagic fever
Dengue virus 1	Dengue virus 1	11053	no rank	Anthropod tranmitted/febrile tropical
Dengue virus 2	Dengue virus 2	11060	no rank	Anthropod tranmitted/febrile tropical
Dengue virus 3	Dengue virus 3	11069	no rank	Anthropod tranmitted/febrile tropical
Dengue virus 4	Dengue virus 4	11070	no rank	Anthropod tranmitted/febrile tropical
Eastern equine encephalitis virus	Eastern equine encephalitis virus	11021	species	Encephalitis
Ebola virus	Ebola virus	1570291	no rank	hemorrhagic fever
Enterovirus	Enterovirus	12059	genus	Enteric
Guanarito virus	Guanarito virus	3052307	species	hemorrhagic fever
Hantavirus	Hantavirus	1980442	genus	Respiratory/Cardiopulmonary
Hendra henipavirus	Hendra henipavirus	3052223	species	Encephalitis
Hepatitis A virus	Hepatitis A virus	12092	species	Bloodborne
Hepatitus B virus	Hepatitus B virus	10407	species	Bloodborne
Hepatitus C virus	Hepatitus C virus	3052230	species	Bloodborne
Hepatitus E virus	Hepatitis E virus/Hepeviridae	291484	family	Bloodborne
Human Immunodeficiency Virus 1	Human Immunodeficiency Virus 1	11676	species	Bloodborne

Human Immunodeficiency Virus 2	Human Immunodeficiency Virus 2	11709	species	Bloodborne
Influenza A Virus	Influenza A Virus	11320	no rank	Respiratory/Cardiopulmonary
Influenza B Virus	Influenza B Virus	11520	no rank	Respiratory/Cardiopulmonary
Japanese encephalitis virus	Japanese encephalitis virus	11072	no rank	Encephalitis
Junin virus	Junin virus	2169991	species	hemorrhagic fever
Kyasanur Forest disease virus	Kyasanur Forest disease virus	33743	no rank	hemorrhagic fever
Lassa fever virus	Lassa mammarenavirus	3052310	species	hemorrhagic fever
Lujo hemorrphagic fever virus	Lujo mammarenavirus	3052314	species	hemorrhagic fever
Machupo virus	Machupo mammarenavirus	3052317	species	hemorrhagic fever
Marburg virus	Marburg marburgvirus	3052505	species	hemorrhagic fever
MERS-CoV	MERS coronavirus	1335626	species	Respiratory/Cardiopulmonary
Metapneumovirus	Metapneumovirus	162387	genus	Respiratory/Cardiopulmonary
Monkeypox virus	Monkeypox virus	10244	species	Rash/lesion
Nipah virus	Nipah virus	3052225	species	Encephalitis
Norovirus	Norovirus	142786	genus	Enteric
Omsk hemorrhagic fever virus	Omsk hemorrhagic fever virus	12542	no rank	hemorrhagic fever
Oncolytic human papillomavirus	human papillomavirus	10566	species	Oncogenic
Parainfluenza virus	Human parainfluenza virus Hue-2015	2559896	species	Respiratory/Cardiopulmonary

	Human parainfluenza virus HCM-2015	2559897	species	Respiratory/Cardiopulmonary
	Human respirovirus 1	12730	no rank	Respiratory/Cardiopulmonary
	Human orthorubulavirus 2	2560525	no rank	Respiratory/Cardiopulmonary
	Human respirovirus 3	11216	no rank	Respiratory/Cardiopulmonary
	Human parainfluenza virus 4a	11224	no rank	Respiratory/Cardiopulmonary
	Human parainfluenza virus 4b	11226	no rank	Respiratory/Cardiopulmonary
	Parainfluenza virus 5	2905673	no rank	Respiratory/Cardiopulmonary
Parechovirus	Parechovirus	138954	genus	Respiratory/Cardiopulmonary
Parvovirus	Parvovirus	1506574	genus	Respiratory/Cardiopulmonary
Poliovirus	Human poliovirus 1	12080	serotype	Enteric
	Human Poliovirus type 2	12083	serotype	Enteric
	Human poliovirus 3	12086	serotype	Enteric
	wild poliovirus type 3	53259	serotype	Enteric
	recombinant polioviruses	909390	no rank	Enteric
	Polyomavirus sp.	36362	species	Oncogenic
Respiratory syncytial virus	Respiratory syncytial virus	12814	no rank	Respiratory/Cardiopulmonary
Rhinovirus	Rhinovirus A	147711	species	Respiratory/Cardiopulmonary
	Rhinovirus B	147712	species	Respiratory/Cardiopulmonary

	Rhinovirus C	463676	species	Respiratory/Cardiopulmonary
	unclassified rhinovirus	348531	no rank	Respiratory/Cardiopulmonary
Rift Valley fever virus	Rift Valley fever virus	11588	no rank	hemorrhagic fever
Rotavirus	Rotavirus	10912	genus	Enteric
Rubella virus	Rubella virus	11041	no rank	Rash/lesion
Sabia virus	Sabia virus	2907957	no rank	hemorrhagic fever
Salivirus	Salivirus	688449	genus	Enteric
Sapovirus	Sapovirus	95341	genus	Enteric
SARS-COV	Severe acute respiratory syndrome coronavirus	2901879	no rank	Respiratory/Cardiopulmonary
SARS-COV-2	Severe acute respiratory syndrome coronavirus 2	2697049	no rank	Respiratory/Cardiopulmonary
Tick-borne encephalitis virus	Tick-borne encephalitis virus	11084	no rank	Encephalitis
Torque Teno virus	Torque Teno virus	68887	species	Bloodborne
Variola virus	Variola virus	10255	species	Rash/lesion
Venezuelan equine encephalitis virus	Venezuelan equine encephalitis virus	11036	species	Encephalitis
West Nile virus	West Nile virus	11082	no rank	Anthropod tranmitted/febrile tropical
Western equine encephalitis virus	Western equine encephalitis virus	11039	species	Encephalitis
Yellow fever virus	Yellow fever virus	11089	no rank	Anthropod tranmitted/febrile tropical
Zika virus	Zika virus	64320	no rank	Anthropod tranmitted/febrile tropical

Table S6. See excel file

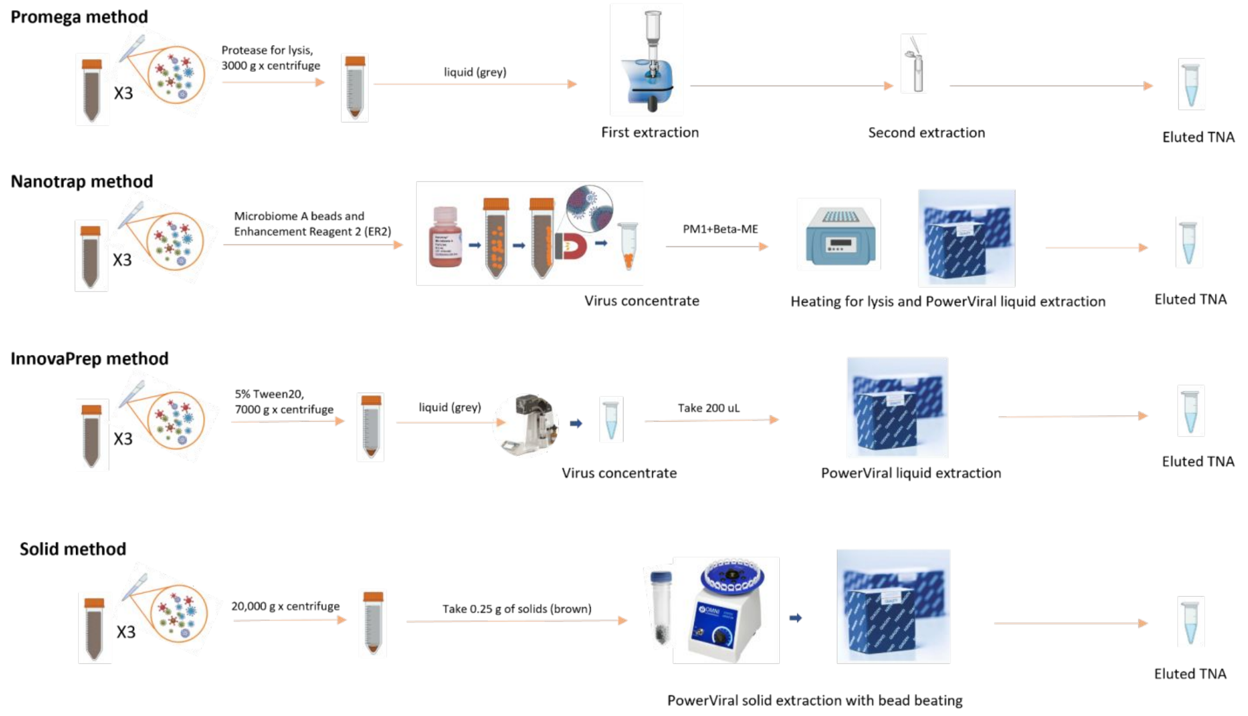

Figure S1. Schematic description of key steps in each concentration and extraction method.

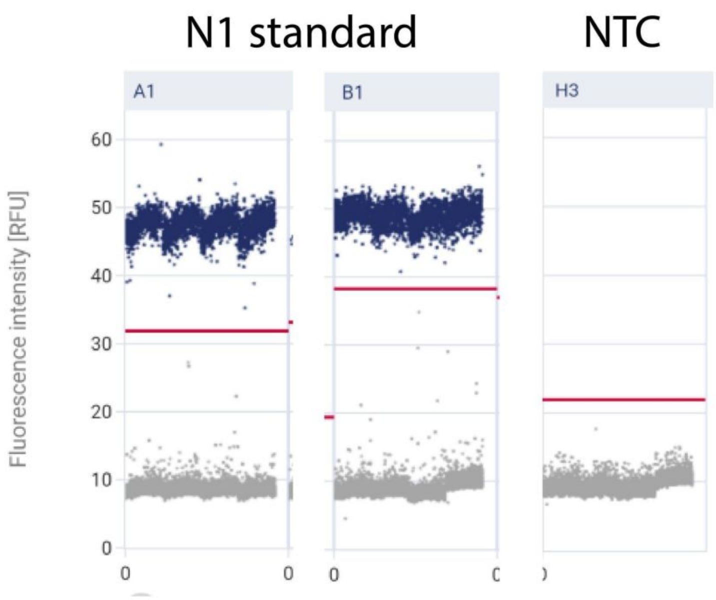

Figure S2. Examples of partition fluorescence plots of dPCR positive and negative control for the CDC N1 assay for SARS-CoV-2.

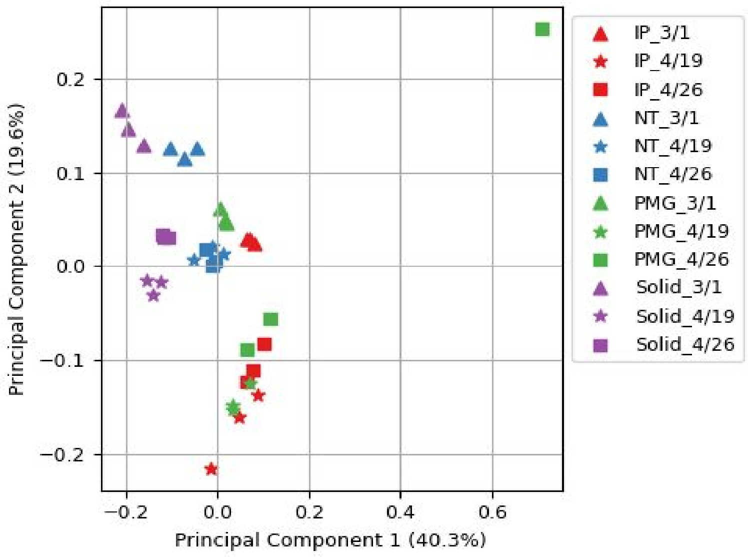

Figure S3. Clustering of all samples by PCoA plot based on the calculated MASH distance of virus sequences classified by Centrifuge. PMG_4/26_2 sample exhibited distinct properties as compared to the other two biological replicates.

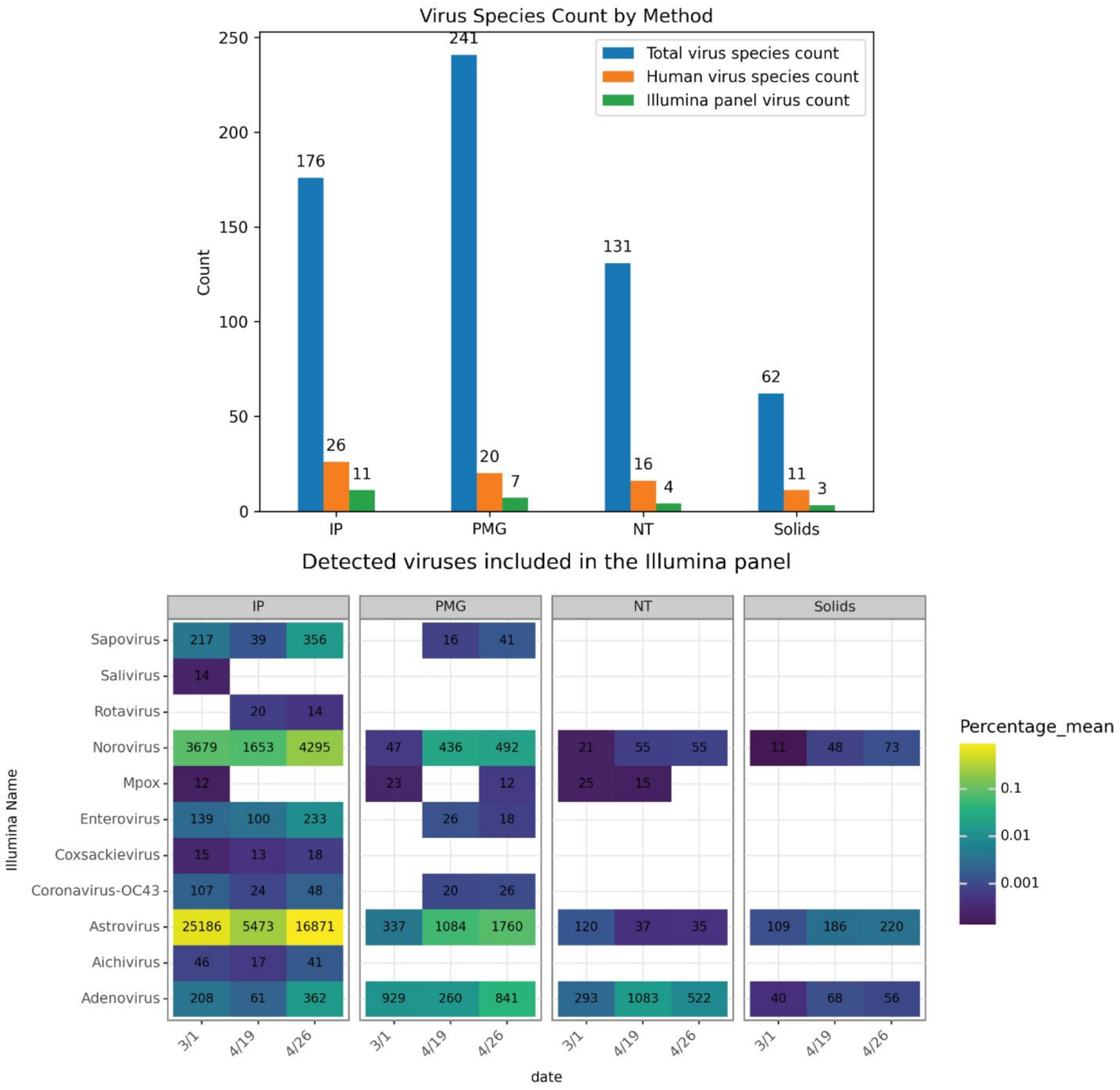

Figure S4. (a) The richness of detected virus species at the species level, encompassing total viruses, human viruses, and those targeted by the Illumina VSP panel. The richness was calculated by counting the total unique taxIDs assigned to species levels in each method; (b) The percentages of the detected viruses included in the VSP panel in total unique reads, filtered by counts exceeding 10 reads. Text in each cell indicates the average read counts assigned to the virus for each sample.

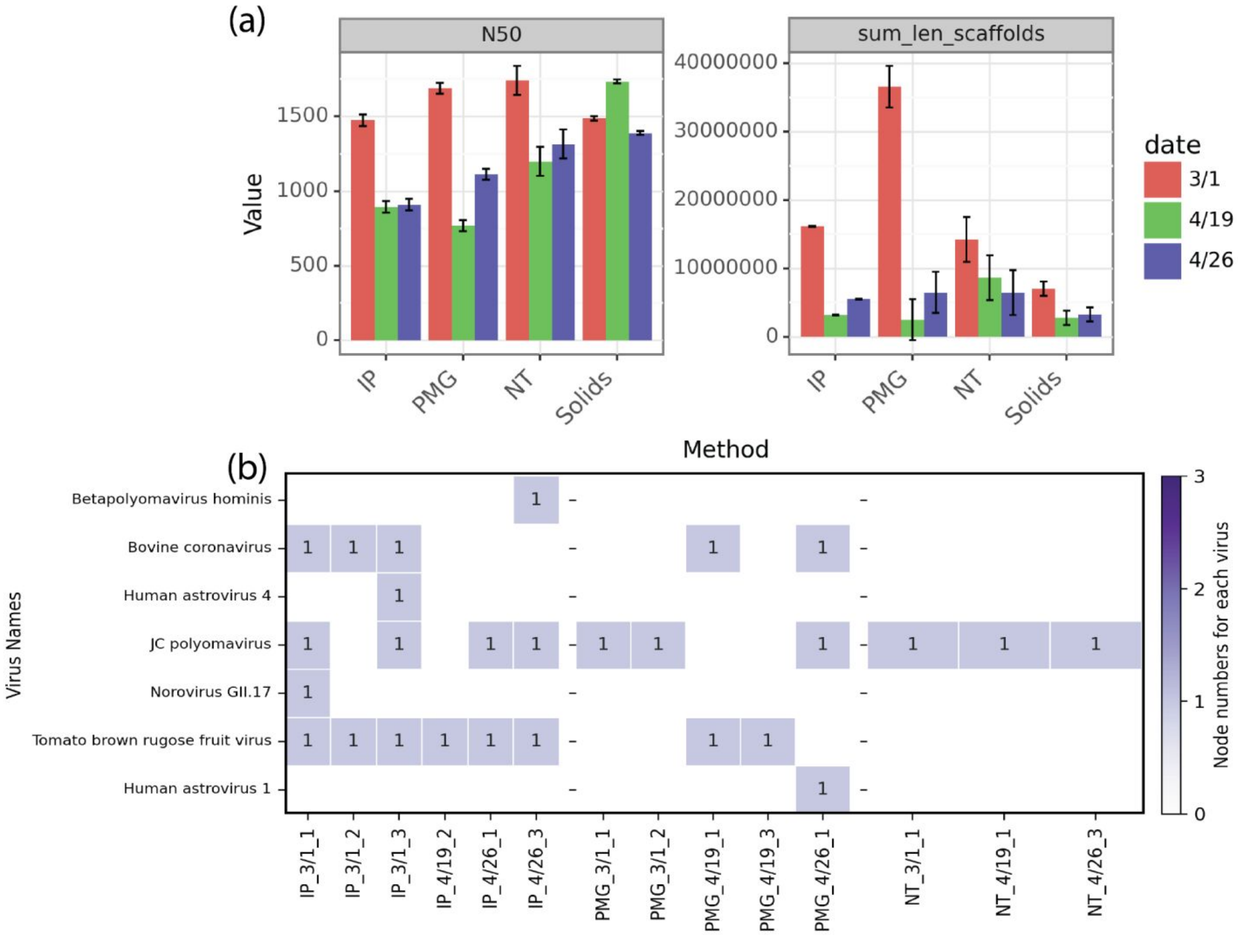

Figure S5. (a) Assessment of assemblies from each method. The assembly quality is based on N50 and the total assembly length. (b) Count of near-complete virus genomes assembled after applying strict criteria (>1000 bp, >10 average coverage depth, >70% coverage breadth of the complete genome, >80% identity, >90% alignment/query length, best hits for each scaffold, and matching complete genomes) to both scaffolds and BLASTn results. Samples with no recovered near-complete virus genomes are not shown.

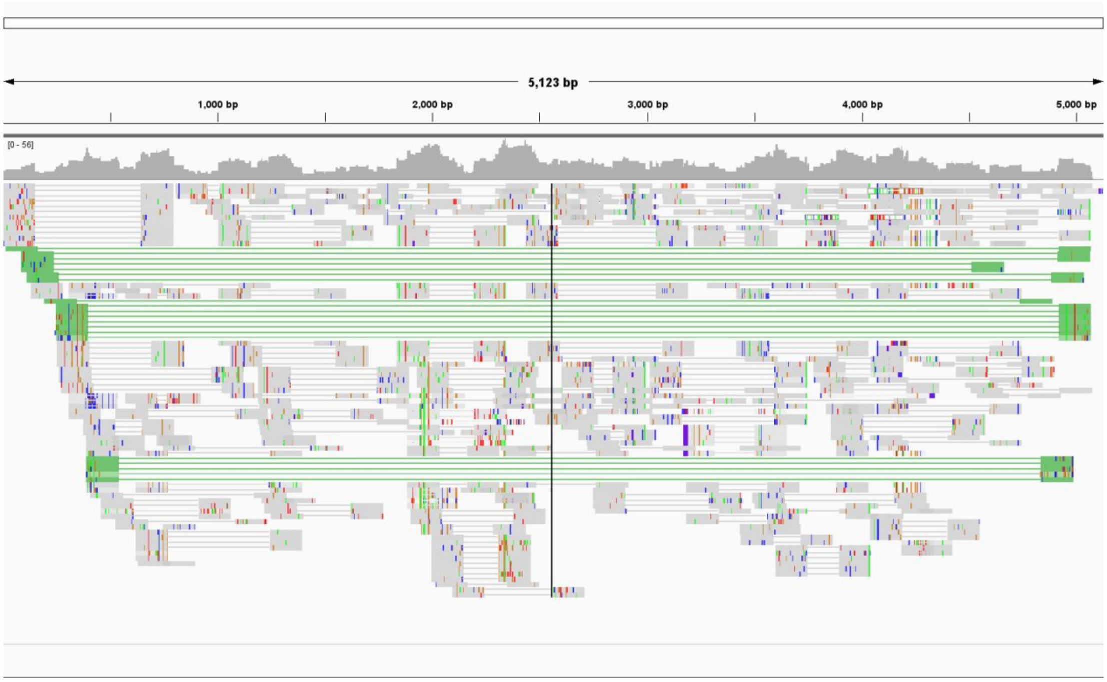

Figure S6. Representative assembly visual inspection by Integrative Genomics Viewer (IGV). The reads were mapped to the assembled putative JC polyomavirus scaffolds (NODE_58_length_5177_cov_12.765912||full) from the PMG_426_1 sample. Both coverage and alignment tracks were shown with mismatches. All reads were colored by pair orientation and shaded by mapping quality high. The coverage allele frequency threshold was set to 0.2. Note that the genome is circular, resulting in read pairs with mates mapping to the 5' and 3' ends when viewed linearly.

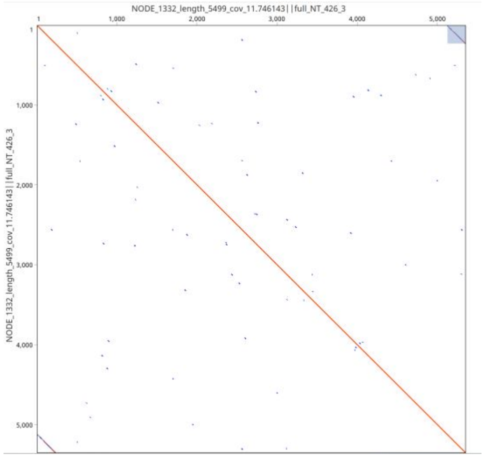

Figure S7. Dotplots of assembled putative JC polyomavirus scaffolds with repeated regions at the beginning and the end of the sequence (see gray box selected in upper right corner). Plot shown represents the sequence of NODE_1332_length_5499_cov_11.746143||full from NT_426_3 sample.

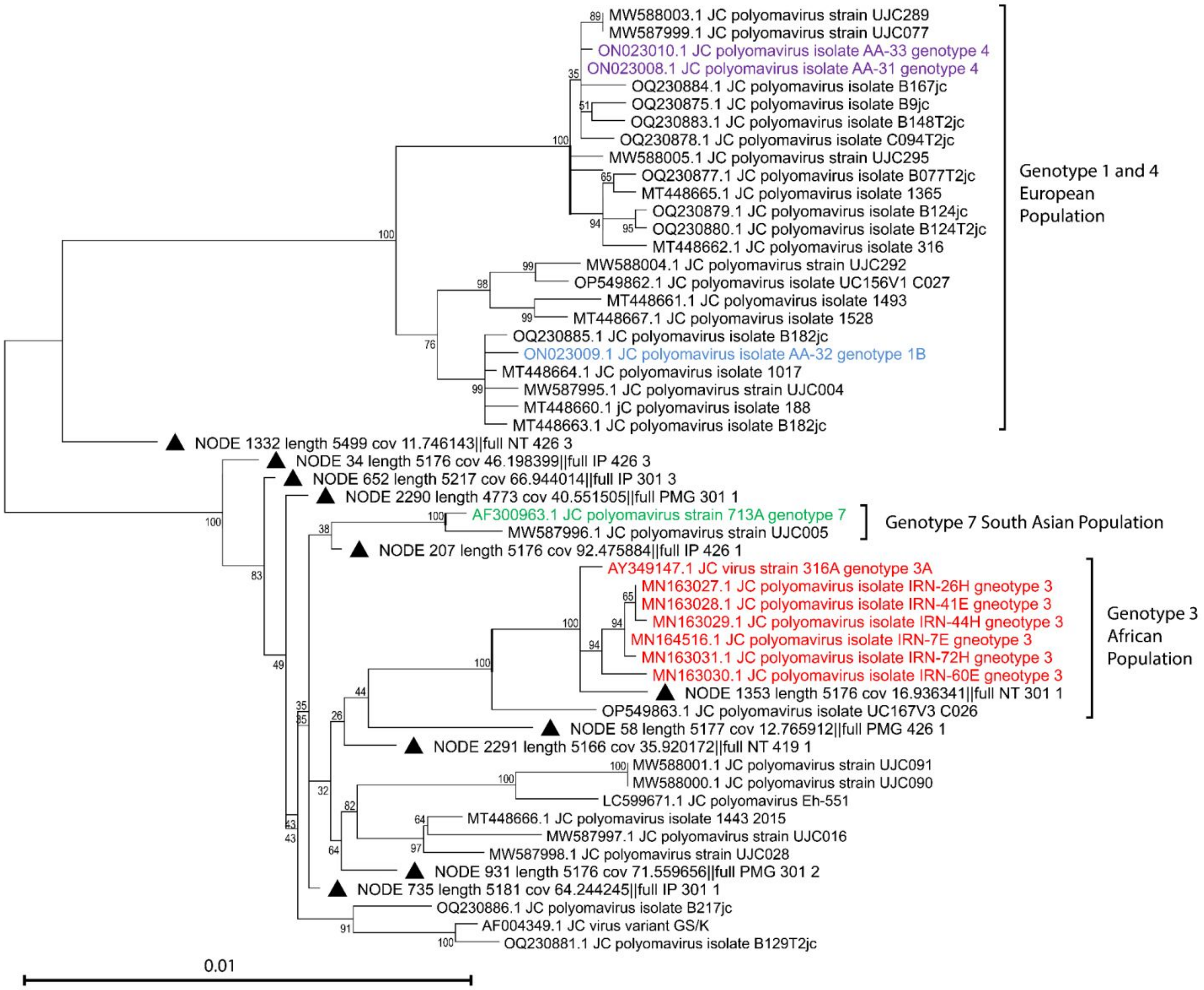

Figure S8. Maximum likelihood phylogenetic tree of assembled JC polyomavirus genomes. The tree was generated using the Maximum Likelihood method, and the best model was selected by ModelFinder (iqtree): Tamura-Nei (TN) + F (Empirical base frequency) + R2 (Free rate model with 2 categories). Node support values, indicating the percentage of trees in which associated taxa clustered together, were obtained from 1000 bootstraps. This analysis included 56 unique nucleotide sequences, with 4168 columns in the final alignment. Reference genomes were colored based on subtypes records on NCBI. Scaffolds from wastewater assemblies are indicated with triangles.

Supplementary methods

Virus-host classification and validation: To determine putative virus host assignments for each read (Figure 2c and 2d), the NCBI taxonomy database, which includes virus host information, was queried with the NCBI taxonomy ID (taxID) of the best hit given by Recentrifuge, and the results were manually inspected. Reads classified as bacteriophage sp. (taxID 38018) and uncultured phage (taxID 278008) were found to have host assigned as "bacteria|human|invertebrates|vertebrates" and "bacteria|fungi|human|invertebrates", respectively. The host for these reads was re-assigned as "bacteria". Additionally, the uncultured viruses (taxID 340016) and circular genetic element sp. (taxID 2202954) were originally assigned to "bacteria|human|invertebrates|land plants|protozoa" and "invertebrates|vertebrates", respectively. The host for these reads was re-assigned as "unknown". Lastly, reads classified as bovine coronavirus were assigned within NCBI as corresponding to the "human|vertebrate" host category and were removed before virus-host analysis.

DNA and RNA virus classification: The comparison of DNA and RNA viruses was conducted at the virus kingdom level based on taxonomic classification results. Six virus kingdoms, namely Orthornavirae, Heunggongvirae, Loebvirae, Sangervirae, Shotokuvirae, and Bamfordvirae, were identified across all methods. These kingdoms were further classified into RNA (Orthornavirae), double-strand DNA (Bamfordvirae and Heunggongvirae), and single-strand DNA viruses (Loebvirae, Sangervirae, Shotokuvirae), following the realms outlined by the International Committee on Taxonomy of Viruses (ICTV).

Human virus species classification: To focus specifically on human viruses, the identification of all human viruses (section 3.3) occurred at the species level based on NCBI taxonomy. Species-level richness was determined by counting the unique human virus species, with a cutoff of > 10 classified reads applied to discard low-abundance viruses (Figure S4a and 4b). Some reads were assigned to species-level NCBI taxIDs that paradoxically lacked clear species-level taxonomy names in the database. The unclassified species includes Human enterovirus (taxID: 1193974), Kobuvirus sp. (taxID: 2094719), Picobirnavirus sp. (taxID: 1907787), Mamastrovirus sp. (taxID: 1912147), Human astrovirus. (taxID:1868658). To denote that these may not represent true species but rather a combination of several unclassified species assigned to the same taxID in NCBI, "unclassified" was appended to the species name.

Phylogenetic analysis of JC polyomavirus: Scaffolds representing near-complete genomes for JC polyomavirus were collected for phylogenetic analyses. Potential assembly errors were inspected by mapping reads to assembled scaffolds using bowtie2 and visualizing with the Integrative Genomics Viewer (IGV v 2.16.2) 39. No assembly errors were detected, and representative mappings are shown in Figure S6. Given the circularity of the JC polyomavirus genome, assemblies were also examined in Geneious 40, and repeated regions at the beginning and the end of the sequences were trimmed before the alignment (Figure S7). Multiple sequence alignment was performed by MUSCLE 41 (v3.8.31) with all trimmed scaffolds, all JC polyomavirus reference genomes from NCBI GenBank released within two years (n = 39), and the best-hit results from BLASTn for each scaffold. The alignment was inspected in Geneious (v 2023.2.1) to

identify a common starting point, and all 10 scaffolds were recircularized to this point. The recircularized scaffolds were queried against NCBI again to identify new best-hit reference genomes, which may have changed due to genome curation. The final dataset included these curated genomes, new best-hits, and the 39 JC polyomavirus references. Alignment was performed with MUSCLE followed by GBlocks 42 (v0.91b) to identify informative regions. IQ-Tree (v2.2.2.6) 43 was used to select the best-fit substitution model: Tamura Nei model with equilibrium base frequency, plus free rate model with 2 categories (TN+F+R2). After generating the maximum likelihood tree with branch support by 1000 ultrafast bootstrap, MEGA 11.0 44 was used to visualize the final tree.
